# Supplementary material for: Variable bites and dynamic populations; new insights in Leishmania transmission
Source: PLoS Negl Trop Dis. 2021 Jan 25;15(1):e0009033. doi: 10.1371/journal.pntd.0009033 (PMC7861551; doi:10.1371/journal.pntd.0009033)
Supplement: S1 Table — The skin heterogeneity and mean skin parasite burden values for RAG mice 1-18 used throughout our simulations, as originally calculated by Doehl et al. [A]: Values derived from Doehl et al. [7]. (PDF) [file pntd.0009033.s001.pdf]

Mouse	Mean Skin Parasite Burden	K Value	Source
RAG 1	1.2697	$1 * 10^{3.2}$	[A]
RAG 2	0.9156	$1 * 10^{3.1}$	[A]
RAG 3	1.2474	$1 * 10^{3.2}$	[A]
RAG 4	3.296	$1 * 10^{3}$	[A]
RAG 5	0.9523	$1 * 10^{3}$	[A]
RAG 6	0.7229	$1 * 10^{4}$	[A]
RAG 7	0.6561	$1 * 10^{5}$	[A]
RAG 8	0.8124	$1 * 10^{5}$	[A]
RAG 9	0.7222	$1 * 10^{4}$	[A]
RAG 10	0.9081	$1.057 * 10^{4}$	[A]
RAG 11	0.7723	$6.2977 * 10^{3}$	[A]
RAG 12	1.1523	$9.424 * 10^{3}$	[A]
RAG 13	1.0874	$8.6053 * 10^{6}$	[A]
RAG 14	1.2146	$6.7356 * 10^{4}$	[A]
RAG 15	0.5017	$2.1384 * 10^{4}$	[A]
RAG 16	1.2758	$1.6032 * 10^{4}$	[A]
RAG 17	1.2462	$1.1973 * 10^{5}$	[A]
RAG 18	1.644	$9.315 * 10^{4}$	[A]
