# Supplementary material for: Variable bites and dynamic populations; new insights in Leishmania transmission
Source: PLoS Negl Trop Dis. 2021 Jan 25;15(1):e0009033. doi: 10.1371/journal.pntd.0009033 (PMC7861551; doi:10.1371/journal.pntd.0009033)
Supplement: S1 Method — (PDF) [file pntd.0009033.s002.pdf]

Parameterisation of Model A

The basic model (Model A) produced in this study focuses on three promastigotes stages, nectomonads, leptomonads and metacyclics. Differential equation based models were produced based on the lifecycle described by Rogers *et al* [1]. This method assumes that the parameters used for rates are all constant. Parameterisation was achieved by fitting data from Rogers *et al* to the model. Data was collected from Figure 1 of this paper using the digitize function in R as this data wasn't readily available [1, 2]. The digitize function is used to manually collected data from plots. The data collected were the total number of parasites in the sand fly and the percentage of each promastigote stage (nectomonad, leptomonad, metacyclic) present in the sand fly over a course of 10 days. This was then used to calculate the number of each promastigote stage. This data was then exported into MATLAB where the function "lsqcurvefit" was used to produce the best fitting parameter values. The quality of fit was assessed via an R^2 value, which defines the proportion of variation that is explained by a model. A high R^2 is indicative of a good fit where as a low R^2 is indicative of a poor fit.

References

1. Rogers ME, Chance ML, Bates PA, The role of promastigote secretory gel in the origin and transmission of the infective stage of Leishmania mexicana by the sandfly Lutzomyia longipalpis. Parasitology. 2002 124:495-507

2. Poisot T, The digitize package: extracting numerical data from scatterplots. R J. 2011 3(1):25-26
