# Supplementary material for: Variable bites and dynamic populations; new insights in Leishmania transmission
Source: PLoS Negl Trop Dis. 2021 Jan 25;15(1):e0009033. doi: 10.1371/journal.pntd.0009033 (PMC7861551; doi:10.1371/journal.pntd.0009033)
Supplement: S2 Method — (PDF) [file pntd.0009033.s003.pdf]

Bite Mechanics

In order to represent a 'patchy' environment for sandflies to draw parasites from, bite loads were generated from a negative binomial distribution using the 'nbinrnd' function in MATLAB. This function outputs a random value from a negative binomial distribution. This takes the following inputs: P (Probability of a positive result, in this case probability that a sand fly will ingest parasites following a bite) and R (the number of successes required). R and P are defined as follows:

$$R = \frac{\mu_N^2}{\sigma_N^2 - \mu_N} \qquad P = \frac{\mu_N}{\sigma_N^2}.$$

where:

$$\mu_N = P_B V_{BM} \qquad \sigma_N^2 = (\mu_N)(1 + \frac{\mu_N}{k})$$

Since this model starts from nectomonads, the number of amastigotes had to be converted to nectomonads. The number of nectomonads is approximately three times greater than the number of amastigotes [1], hence the number of amastigotes was multiplied by three to estimate the number of nectomonads.

References

1. Rogers ME, Chance ML, Bates PA, The role of promastigote secretory gel in the origin and transmission of the infective stage of Leishmania mexicana by the sandfly Lutzomyia longipalpis. Parasitology. 2002 124:495-507
