# Supplementary material for: Variable bites and dynamic populations; new insights in Leishmania transmission
Source: PLoS Negl Trop Dis. 2021 Jan 25;15(1):e0009033. doi: 10.1371/journal.pntd.0009033 (PMC7861551; doi:10.1371/journal.pntd.0009033)
Supplement: S3 Method — (PDF) [file pntd.0009033.s004.pdf]

Population Sink Model Definitions

In order to assess the sensitivity of our models to the assumption that we have 100% efficiency in converting between lifecycle stages, we present an adapted form of the model which incorporates a population sink at each lifecycle stage. For simplicity we assume that the population sinks are some constant death rate per parasite per day (or equivalently a loss of parasite differentiation capacity) and that this rate does not vary between lifecycle stages. With the inclusion of the population sinks, Model A is now:

$$\frac{dN}{dt} = -\alpha N - \gamma N \quad (1)$$

$$\frac{dL}{dt} = \alpha N + rL\left(1 - \frac{N+L+M}{C}\right) - sL - \gamma L \quad (2)$$

$$\frac{dM}{dt} = sL - uM - \gamma M \quad (3)$$

where we assume γ to be constant. Model B can be modified similarly. Normal mode is now:

$$\frac{dN}{dt} = -\alpha N - \gamma N \quad (4)$$

$$\frac{dL}{dt} = \alpha N + rL\left(1 - \frac{N+L+M+R}{C}\right) - sL - \gamma L \quad (5)$$

$$\frac{dM}{dt} = sL + vR - uM - \gamma M \quad (6)$$

$$\frac{dR}{dt} = qR\left(1 - \frac{N+L+M+R}{C}\right) - vR - \gamma R \quad (7)$$

and dedifferentiation mode is:

$$\frac{dM}{dt} = sL - gM - uM - \gamma M \quad (8)$$

$$\frac{dR}{dt} = qR\left(1 - \frac{N+L+M+R}{C}\right) + gM - \gamma R \quad (9)$$

We shall consider two different exemplar values of γ. To represent a small population sink, we consider $\gamma = 0.05$. For a larger sink, we choose $\gamma = 0.15$.
