# Supplementary figures and images for: Variable bites and dynamic populations; new insights in Leishmania transmission
Source: PLoS Negl Trop Dis. 2021 Jan 25;15(1):e0009033. doi: 10.1371/journal.pntd.0009033 (PMC7861551; doi:10.1371/journal.pntd.0009033)

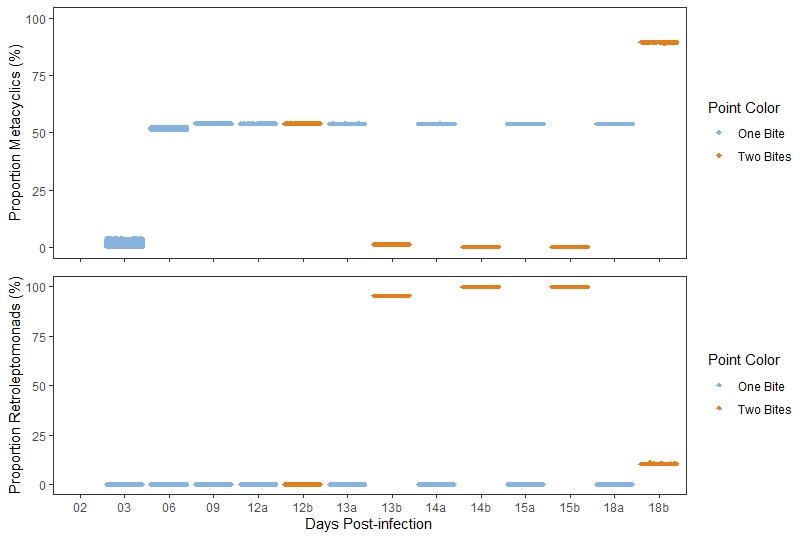

Supplement: S1 Fig — Comparison of the proportions of metacyclics (top) and retroleptomonads (bottom) at specific days throughout the lifespan of the simulated flies. Blue represents flies that bite only at day 0, orange represents flies that bite at day 12. The two categories are combined prior to day 12. (TIF) [file pntd.0009033.s006.tif]

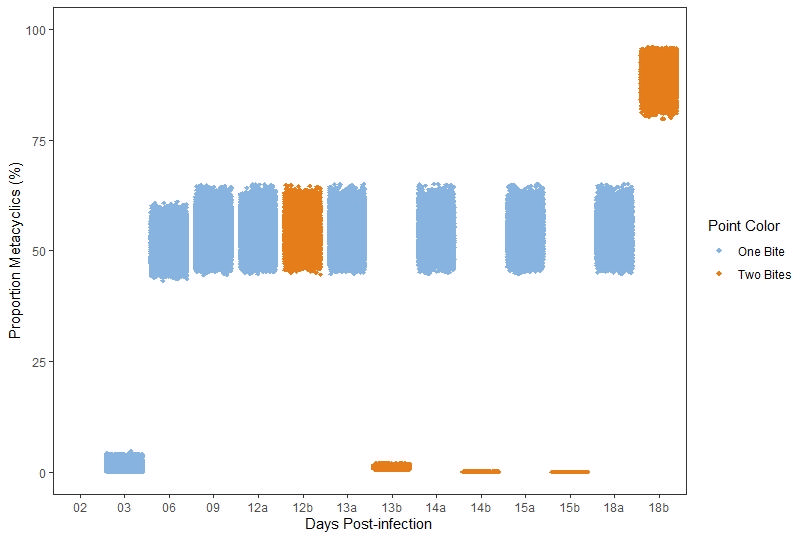

Supplement: S2 Fig — Number of metacyclics within the sand flies at specific days, with all parameters randomised prior to the start of each simulation. Parameters lie within 10% of the default value (Table 1). Blue represents flies that bite only a day 0, orange represents flies that bite at day 12. (TIF) [file pntd.0009033.s007.tif]

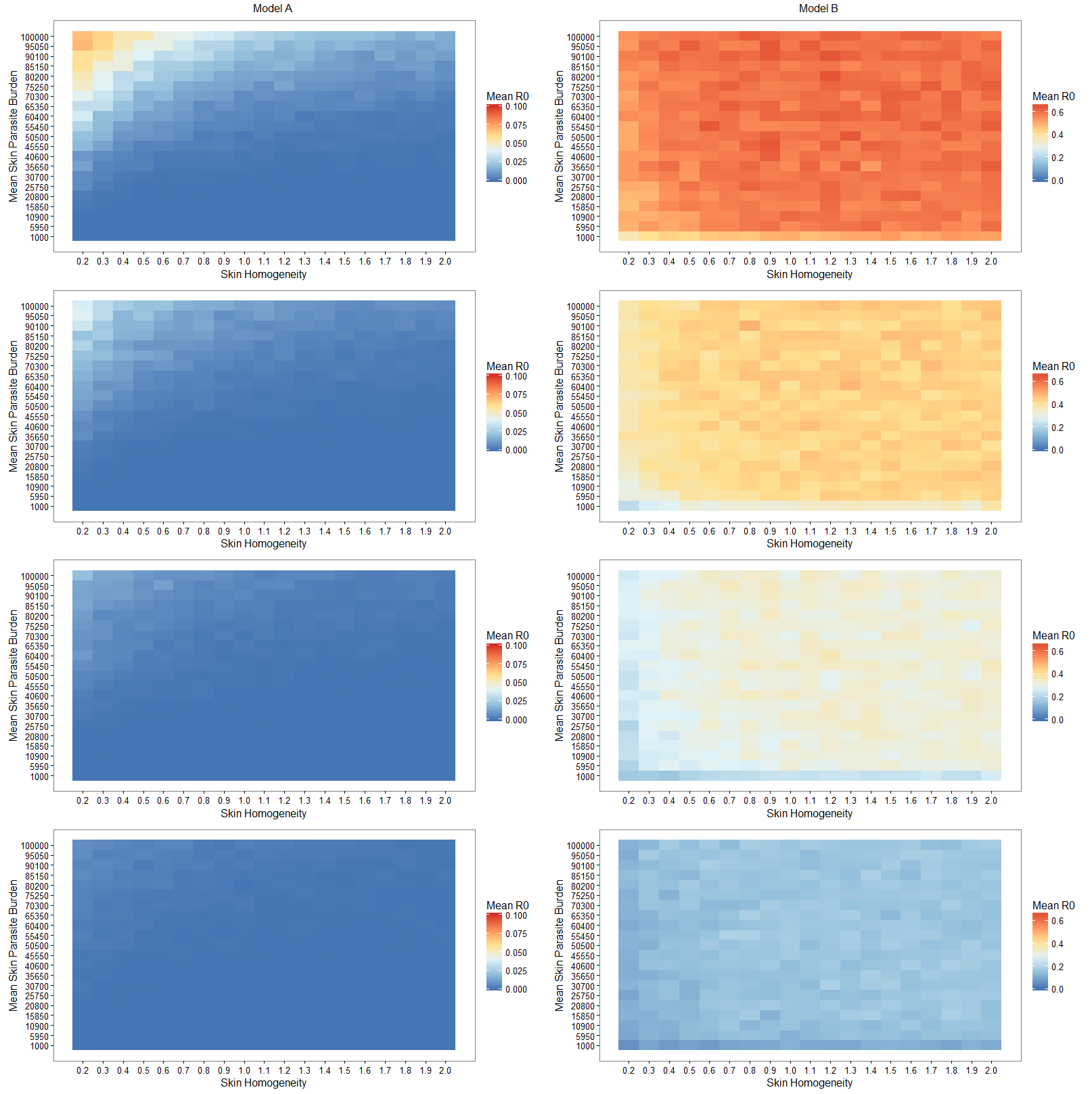

Supplement: S3 Fig — Heatmaps of the Mean R0 for simulated sand flies for both Model A (left half) and B (right half) with 100% (top row), 50% (second row), 25% (third row), and 10% (bottom row) chance of biting an infected host, with the smooth transmission threshold function. (TIF) [file pntd.0009033.s008.tif]

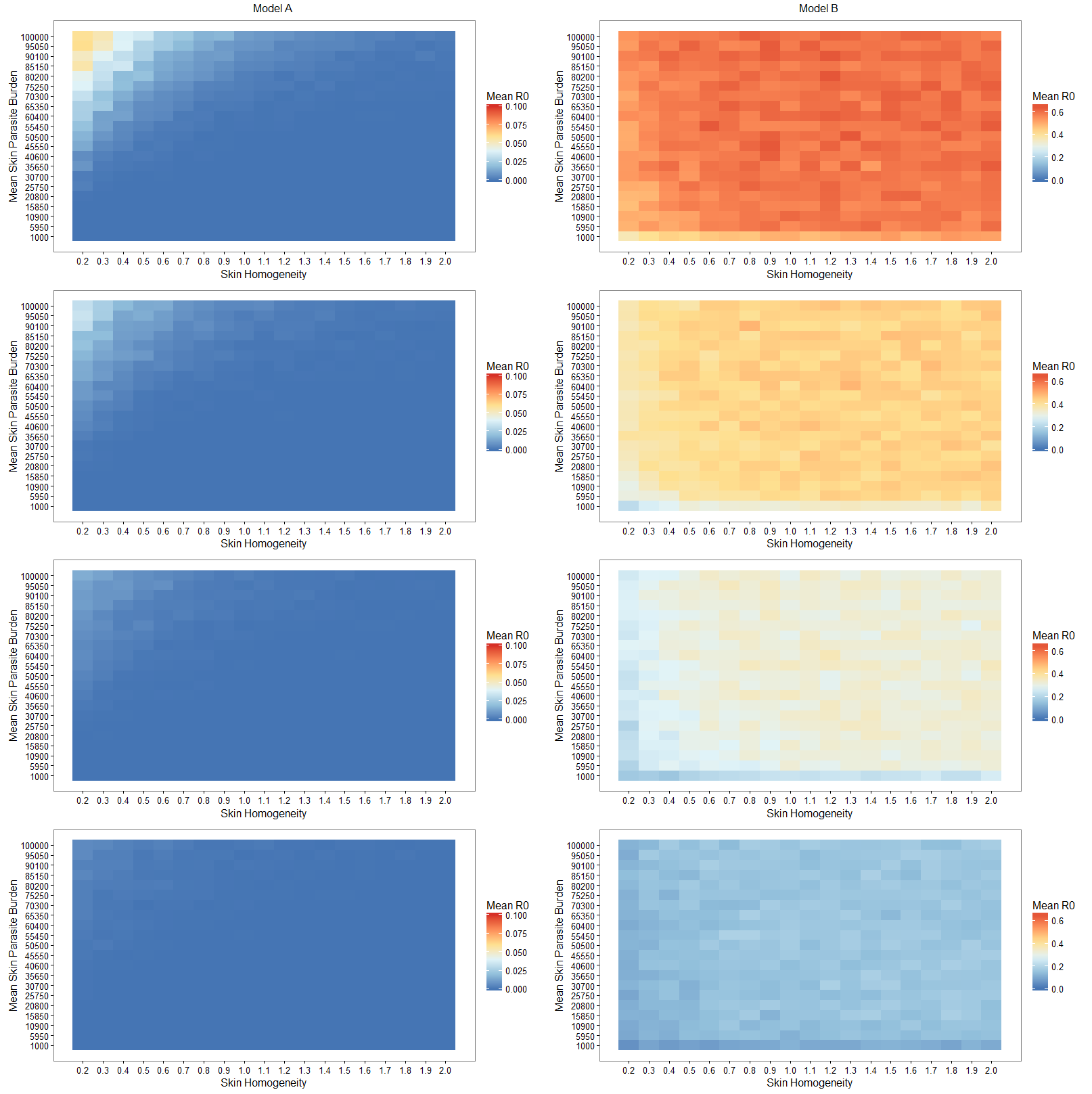

Supplement: S4 Fig — Heatmaps of the Mean R0 for simulated sand flies for both Model A (left half) and B (right half) with 100% (top row), 50% (second row), 25% (third row), and 10% (bottom row) chance of biting an infected host, with the binary transmission threshold. (TIF) [file pntd.0009033.s009.tif]

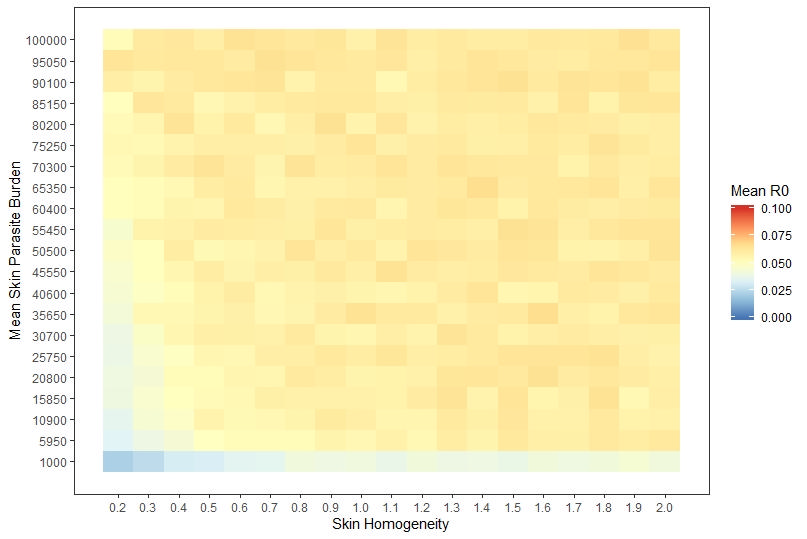

Supplement: S5 Fig — Heatmap of the Mean R0 for simulated sand flies in Model B with 100% chance of biting an infected host and with lifespans restricted to 20 days, with the binary transmission threshold. (TIF) [file pntd.0009033.s010.tif]

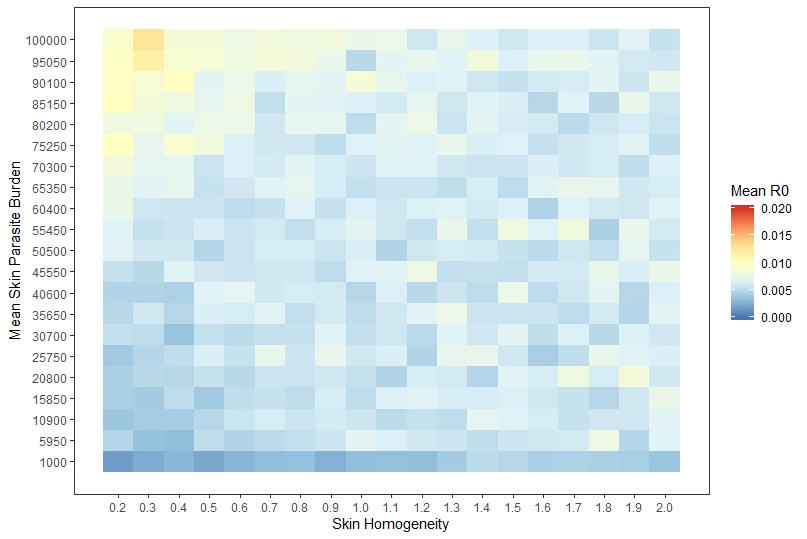

Supplement: S6 Fig — Heatmap of the Mean R0 for simulated sand flies in Model B with 100% chance of biting an infected host and with lifespans restricted to 15 days, with the binary transmission threshold. (TIF) [file pntd.0009033.s011.tif]

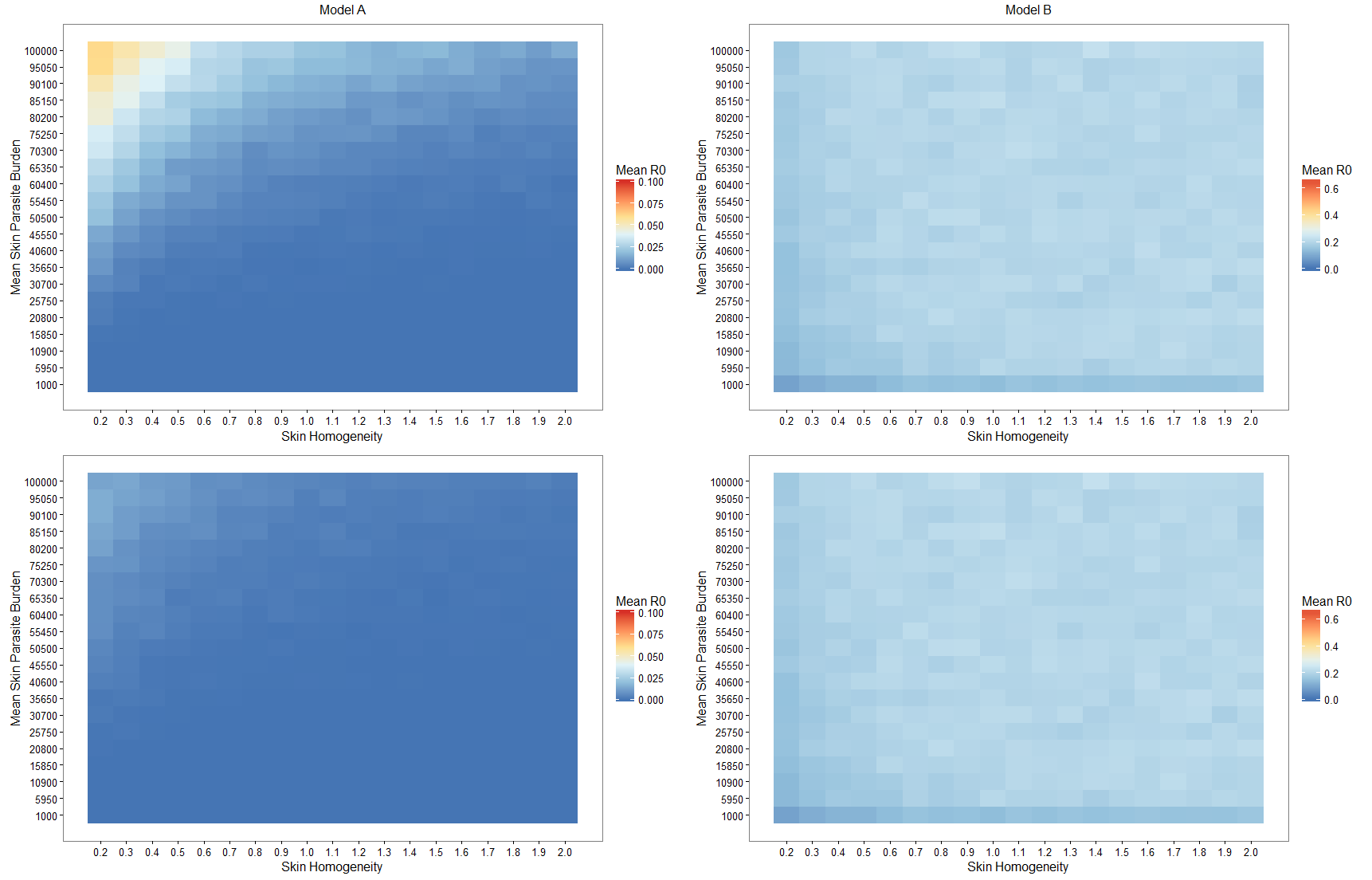

Supplement: S7 Fig — Heatmaps of Mean R0 for simulated sand flies for both Model A (left half) and B (right half) with 100% (top row) or 25% (bottom row) chance of biting an infected host, with a smooth transmission threshold. After infection, sand flies receive a 20% reduction to their remaining lifespan. (TIF) [file pntd.0009033.s012.tif]

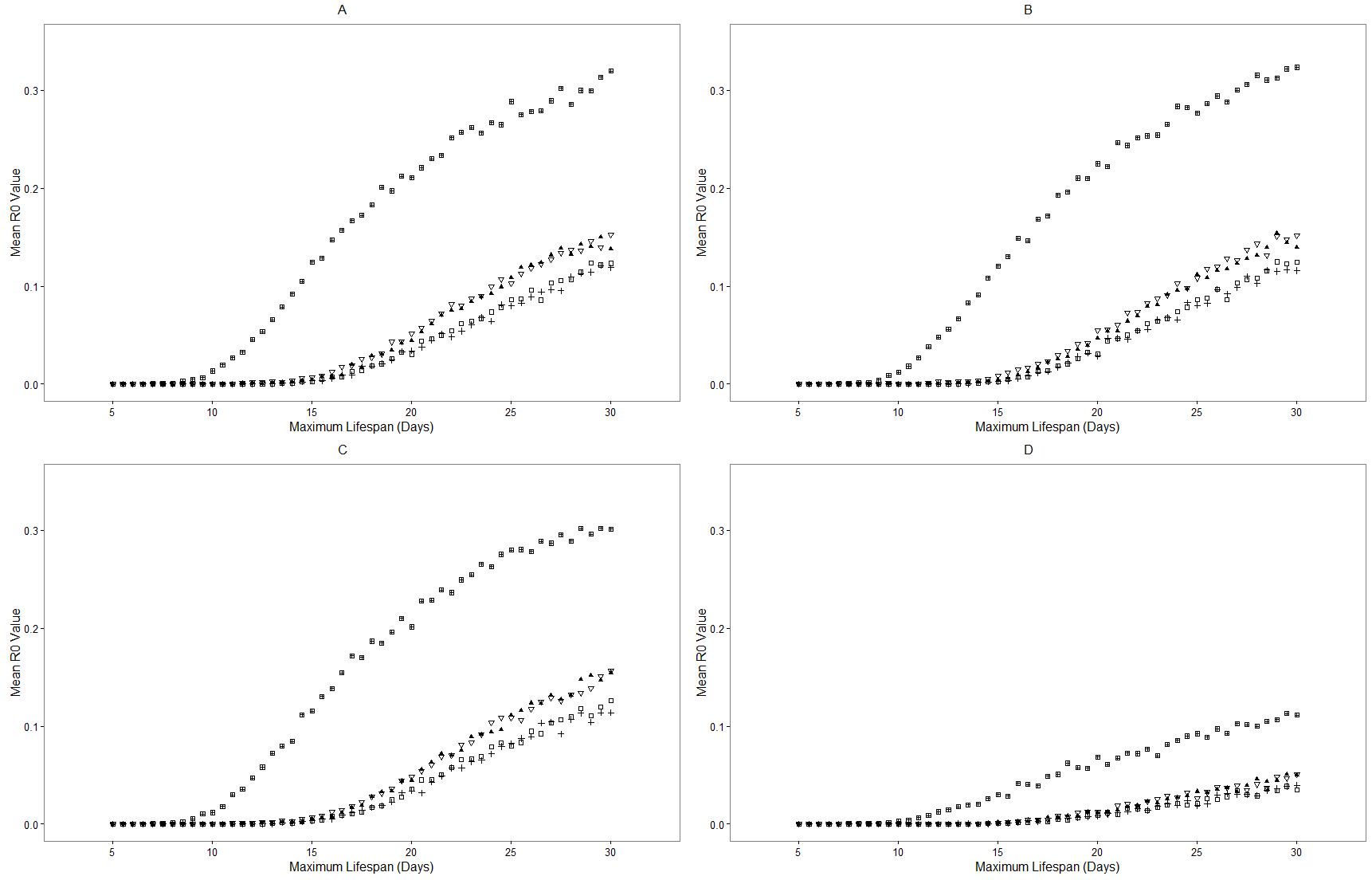

Supplement: S8 Fig — Mean R0 against maximum lifespan for a representative subsample of RAG mice. A) Full model adapted from Fig 4c. B) Full model, but with no carrying capacity. C) Full model, but with additional small population sinks. D) Full model, but with larger population sinks. (TIF) [file pntd.0009033.s013.tif]
