# Supplementary material for: Variable bites and dynamic populations; new insights in Leishmania transmission
Source: PLoS Negl Trop Dis. 2021 Jan 25;15(1):e0009033. doi: 10.1371/journal.pntd.0009033 (PMC7861551; doi:10.1371/journal.pntd.0009033)
Supplement: Attachment — Submitted filename: PNTD-D-20-01530Review.pdf [file pntd.0009033.s015.pdf]

This manuscript addresses two very interesting new biological findings regarding the Leishmania parasite life cycle (skin heterogeneity and retroleptomonad promastigote stages) and try to assess their epidemiologic importance in transmission. The assessment is carried on by the introduction of a population dynamics model where the trade-offs implied by the new mechanism are made explicit. The discussion that follows looks plausible and well supported by the algebraic steps taken along the way.

In general, I am pleased with the work and read it with interest. However, what I miss most in this work is a more thorough assessment of the sensitivity of the main conclusions to the main assumptions ("differentiation between parasite life stages occurs at 100% efficiency and that there is a single globally applied carrying capacity"). Also, I feel uncomfortable with passages such as this one in line 111: "we present expressions bounding implied disease transmission probabilities in a range of hypothetical scenarios". My distress stems from the fact that the authors do not formulate a true probabilistic model since there is just one source of randomness (N0 in line 117) that propagates deterministically along with the subsequent steps. I would rate the treatment of uncertainty in this work as rather unsatisfactory.

Therefore, given my comments on the previous paragraph, I would encourage the authors to address the robustness of their findings as well as the source of uncertainties as topics that could improve their discussion section.
