# Supplementary material for: Variable bites and dynamic populations; new insights in Leishmania transmission
Source: PLoS Negl Trop Dis. 2021 Jan 25;15(1):e0009033. doi: 10.1371/journal.pntd.0009033 (PMC7861551; doi:10.1371/journal.pntd.0009033)
Supplement: Attachment — Submitted filename: Review report.docx [file pntd.0009033.s014.docx]

Review report:

The authors investigate the relationship of mean R0 for an infective sand fly that takes multiple bloodmeals and heterogeneity of *Leishmania* distribution in host skin using mathematical modelling. As expected, the authors show that sand flies that feed multiple times and are long-lived have a higher mean R0 and transmit more efficiently due to amplification of infectious stage metacyclics via the multiplicative, recently described, retroleptomonad stage. The authors also show that patchiness in the distribution of parasites in host skin becomes less relevant for successful transmission by infected flies that take multiple blood meals. Additionally, the threshold for the number of parasites needed to generate a successful infection after being taken up by a sand fly is reduced. Though the model and simulations produce somewhat expected outcomes, the paper brings two factors, currently viewed as independent findings in recent publications, together and simulates how they influence one another, a relevant and pertinent advance to our grasp of the complexity of *Leishmania* transmission under natural conditions.

There are several points that the authors need to address:

Main points:

The paper needs to standalone. Though the authors base their model on findings of two recent publications, only referring to previously published data for specific information on which they base their own data is confusing and requires that the reader is familiar with the details of these other publications to understand the current one. The authors should provide a description of the source data they are using in such a way that it clarifies their significance in the model and relevance of output data. Eg. In line 98, the authors refer to mice 10-18 from Doehl et al without further information; in Lines 103-104, how would the reader know that mouse 13 is heavily infected and mice 10 and 16 had lighter infections? Not clear. The authors do show this using Xs in Figure 4A/B but this needs to be clear for all simulations using similar information. The authors should introduce the characteristics defining patchiness of mice 1-18 used in their analyses within this work and find a way to represent the information in their own paper.

There are some fundamental information that I recommend be mentioned in the introduction so that they are clear from the outset of the paper. It would be pertinent that the authors clarify that in nature the sand fly will likely take a bloodmeal every 5-6 days (sand flies are mostly promiscuous feeders and would feed on most mammals so therefore the source of blood will not be restrictive). Further, transmission in natural conditions only occurs by a sand fly that has taken at least 2 previous bloodmeals. A sand fly will never take a blood meal on day 0 and then take another only on day 12; in reality on day 12 it is likely to be the sand fly’s 3^rd^ bloodmeal. So in the introduction the authors describe Serafim et al’s experimental approach without clarifying it as such though they later model a bloodmeal every 6 days. The authors should also comment that the model for patchy parasite distribution was based on immunodeficient mice that only represent immunosuppressed individuals (as is the case with clinically symptomatic visceral leishmaniasis patients). Parasite patchiness is yet to be shown for asymptomatic immunocompetent infected subjects. These statements clarifies to the reader a more biologically accurate interpretation of the published findings. These clarification do not reflect on the model but are important to set the stage for it.

In line 115 the authors say “when it ingests N0 parasites in the nectomonad life stage”. Though this is a model, I do not think that the authors need to say this. The model should be built on biologically accurate information, sand flies will never ingest nectomonads; however, nectomonads will appear in the sand flies by day 3 and are the majority of the population on day 4, as the authors mention elsewhere in their paper. So, could the authors rephrase the description of the model to take that into consideration? Eg. define N0 as the number of nectomonads present in the fly 4 days post-bloodmeal as a more accurate biological description of the state of the fly.

Did the authors mean to say an additional infected bite scenario in Ln122? The retroleptomonads phenomenon in Serafim et al is observed upon uptake of a subsequent uninfected bloodmeal. If the authors did mean another bite on an infected host, what is the implication of that compared to another uninfected bite which is a more likely scenario than feeding again on an infected host. Also the equation after line 28 will not only be dependent on N0 with another multiplicative constant as you have another ingestion of parasites from the infected bloodmeal at time day 6. Unless the authors did mean ingestion of an ‘uninfected’ blood meal?

The authors provide a mean of 13 days for the life span of the sand fly but do not provide information on the distribution or variance of the sand fly population used for the model. Also, the fly may only get infected on its 2nd or 3rd bloodmeal, so perhaps the authors can refer to lifespans of the simulated flies to “days after the initial infected bloodmeal”.

In the discussion, the authors refer to the importance of PSG in promoting transmission. This is rightly so, however, the influence of PSG is highly dependent on the density of the parasites. PSG is produced by promastigotes and if there are a lower density of parasites, as would occur in flies without the phenomenon of retroleptomonads, the PSG plug will be less well formed, so therefore the frustrated behavior and efficient transmission would also be directly affected by this. Just something to consider.

Minor corrections

Ln35 qualify ‘sand fly with a mature infection’

Ln40 single ~~bite~~ fed sand fly (makes clearer to the reader)

Ln 101:do the authors mean for a fly to be considered “infectious”? A fly is infected if it contains parasites

Fig. 2: “orange represents flies that take a subsequent bite at day 12” or “that bite at days 0 and 12”..

Fig. 2B shows mice ~~10~~9-18

Fig. 4C Again reference to mice from Doehl et al. must be qualified by a description of their status as this paper must be standalone to readers

Ln 181 that a transmission

Ln196 vs Fig. 3 legend: legend says “with 100% (top half) or 25% (bottom half) chance of biting an infected host.” This is not the same as assuming that 100% or 25% of the hosts are infected. Please be consistent in your meaning and assumptions (Ln196).

Ln298 where leishmaniasis is endemic
