# Supplementary material for: Variable bites and dynamic populations; new insights in Leishmania transmission
Source: PLoS Negl Trop Dis. 2021 Jan 25;15(1):e0009033. doi: 10.1371/journal.pntd.0009033 (PMC7861551; doi:10.1371/journal.pntd.0009033)
Supplement: Attachment — Submitted filename: ResponseLetter.docx [file pntd.0009033.s016.docx]

Responses to Reviewer Comments

**Dear Dr. Ribeiro and Dr. Valenzuela,**

**We are grateful to our three reviewers for their insightful and constructive comments and have taken steps to address each of them. We have made substantial changes to our manuscript to incorporate their suggestions as well as minor changes throughout the manuscript to improve its clarity.**

**Below we include our responses to each of the reviewers’ comments, which we have labelled for ease of reference. With our resubmission, we enclose a document with our changes highlighted and an equivalent plain text version. Our comments include references to the location of the relevant changes within the highlighted document and to any relevant supplementary material files.**

**Once again, we would like to thank our reviewers for the time and effort invested in producing feedback that has enabled such improvements to our manuscript, and sincerely hope that our revised manuscript will now prove suitable for publication in PLOS Neglected Tropical Diseases.**

Reviewer 1

1) “The paper needs to standalone. Though the authors base their model on findings of two recent publications, only referring to previously published data for specific information on which they base their own data is confusing and requires that the reader is familiar with the details of these other publications to understand the current one. The authors should provide a description of the source data they are using in such a way that it clarifies their significance in the model and relevance of output data. Eg. In line 98, the authors refer to mice 10-18 from Doehl et al without further information; in Lines 103-104, how would the reader know that mouse 13 is heavily infected and mice 10 and 16 had lighter infections? Not clear. The authors do show this using Xs in Figure 4A/B but this needs to be clear for all simulations using similar information. The authors should introduce the characteristics defining patchiness of mice 1-18 used in their analyses within this work and find a way to represent the information in their own paper.”

**Response: We have added a new table (Table S1) to our supplementary materials that provides details of the parameter combinations calculated by Doehl *et al.* upon which we base our exemplar mice. We now reference this table throughout the manuscript at all points where we rely upon these parameters, including at lines 118-119, line 271, and the captions of Figures 2 and 4.**

2) “It would be pertinent that the authors clarify that in nature the sand fly will likely take a bloodmeal every 5-6 days (sand flies are mostly promiscuous feeders and would feed on most mammals so therefore the source of blood will not be restrictive). Further, transmission in natural conditions only occurs by a sand fly that has taken at least 2 previous bloodmeals. A sand fly will never take a blood meal on day 0 and then take another only on day 12; in reality on day 12 it is likely to be the sand fly’s 3^rd^ bloodmeal. So in the introduction the authors describe Serafim et al’s experimental approach without clarifying it as such though they later model a bloodmeal every 6 days.”

**Response: We have updated our introduction to include a short section clarifying that sand flies are typically opportunistic feeders (lines 9-11), as well as a short additional section discussing it further in our discussion (lines 363, 365 and 366). We also incorporate a short section in our introduction (lines 49-53) discussing how sand flies may not be expected to infect until their third bite.**

3) “The authors should also comment that the model for patchy parasite distribution was based on immunodeficient mice that only represent immunosuppressed individuals (as is the case with clinically symptomatic visceral leishmaniasis patients). Parasite patchiness is yet to be shown for asymptomatic immunocompetent infected subjects. These statements clarifies to the reader a more biologically accurate interpretation of the published findings.”

**Response: We have incorporated a short section in our introduction at lines 30-32 with additional details about the methodology of Doehl *et al.*, namely that they considered infections in immunodeficient mice. We also provide a short discussion at lines 333-337 to highlight that such mice do not represent immunocompetent individuals.**

4) “In line 115 the authors say “when it ingests N0 parasites in the nectomonad life stage”. Though this is a model, I do not think that the authors need to say this. The model should be built on biologically accurate information, sand flies will never ingest nectomonads; however, nectomonads will appear in the sand flies by day 3 and are the majority of the population on day 4, as the authors mention elsewhere in their paper. So, could the authors rephrase the description of the model to take that into consideration? Eg. define N0 as the number of nectomonads present in the fly 4 days post-bloodmeal as a more accurate biological description of the state of the fly.”

**Response: We have updated our definition of N_0_ at lines 138-140 as per the reviewer’s suggestion, and have also added some clarity about our choice of t = 0. We now specify that we assume our model starts at the first nectomonad peak, and that N_0_ is the number of parasites present in the sand fly initially.**

5) “Did the authors mean to say an additional infected bite scenario in Ln122?”

**Response: We did in fact mean an additional uninfected bite (in line with the experimental setup of Serafim *et al.*). We thank the reviewer for identifying this error in the text, and have corrected it accordingly.**

6) “The authors provide a mean of 13 days for the life span of the sand fly but do not provide information on the distribution or variance of the sand fly population used for the model. “

**Response: In addition to stating the distributions upon which we base our sand fly lifespans and inter-bite times, we now also state the corresponding standard deviations and means (at lines 194 and 196 for the lifespan and inter-bite times respectively).**

7) “Also, the fly may only get infected on its 2nd or 3rd bloodmeal, so perhaps the authors can refer to lifespans of the simulated flies to “days after the initial infected bloodmeal””

**Response: We have updated the text at line 199 to clarify that in the scenario used throughout section 1.4 the sand flies are initially uninfected. As the reviewer correctly notes, the sand flies may become infected at any bite during their adult lifespan, or indeed may not live sufficiently long to become infected. However, in section 1.4 we assume that each of our simulated sand flies is a new mature female that has not yet fed at t = 0.**

8) “In the discussion, the authors refer to the importance of PSG in promoting transmission. This is rightly so, however, the influence of PSG is highly dependent on the density of the parasites. PSG is produced by promastigotes and if there are a lower density of parasites, as would occur in flies without the phenomenon of retroleptomonads, the PSG plug will be less well formed, so therefore the frustrated behavior and efficient transmission would also be directly affected by this. Just something to consider.”

**Response: We believe that this point is addressed by the inclusion of Giraud *et al.* (2019) in our discussion as part of our response to Reviewer 3, Point 2.**

9) Ln35 qualify ‘sand fly with a mature infection’

**Response: We have updated line 42 as suggested.**

10) Ln40 single ~~bite~~ fed sand fly (makes clearer to the reader)

**Response: We have updated line 47 to read “a sand fly that has fed only once” for clarity.**

11) Ln 101: do the authors mean for a fly to be considered “infectious”? A fly is infected if it contains parasites

**Response: We are grateful to the reviewer for highlighting a lack of clarity in describing our approach to replicating Doehl *et al.* and have rewritten the section (lines 107-122). The reviewer is correct that sand flies containing any number of parasites could be considered infected. We now refer to sand flies that surpass our chosen threshold of metacyclics as “infectious” as suggested, in order to distinguish them from sand flies which carry very small numbers of metacyclics at day 7 (and which, *in vivo*, would be unlikely to have maintained the infection). It is important to note that our “infectious” sand flies are comparable to those referred to as “infected” by Doehl *et al.***

**Additionally, we have updated the labelling in Fig. 2B to reflect the new definitions.**

12) “Fig. 2: “orange represents flies that take a subsequent bite at day 12” or “that bite at days 0 and 12”..”

**Response: We have updated the caption of Fig. 2 to read “orange represents flies that take a subsequent bloodmeal at day 12”.**

13) “Fig. 2B shows mice ~~10~~9-18”

**Response: We thank the reviewer for highlighting this error in Fig. 2B. In line with the work of Doehl *et al.*, we have updated Fig. 2B to correctly display mice 10-18.**

14) Fig. 4C Again reference to mice from Doehl et al. must be qualified by a description of their status as this paper must be standalone to readers

**Response: We now reference the newly added Table S1 in the caption of Fig. 4. See our response to point 1 for further details.**

15) Ln 181 that a transmission

**Response: We have now corrected this in the text.**

16) Ln196 vs Fig. 3 legend: legend says “with 100% (top half) or 25% (bottom half) chance of biting an infected host.” This is not the same as assuming that 100% or 25% of the hosts are infected. Please be consistent in your meaning and assumptions (Ln196).

**Response: We have updated the text at line 224 to clarify our assumptions. In this section, we assume that our simulated sand flies bite at random from our population without preference. Under this assumption, the chance of biting an infected host is equal to the proportion of the population that is infected.**

17) Ln298 where leishmaniasis is endemic

**Response: We have updated line 369 for clarity, as suggested.**

Reviewer 2

1) “However, what I miss most in this work is a more thorough assessment of the sensitivity of the main conclusions to the main assumptions (“differentiation between parasite life stages occurs at 100% efficiency and that there is a single globally applied carrying capacity”).”

**Response: We agree that a more thorough assessment of the sensitivity of our model would contribute improve the quality of our manuscript. We have run a suite of new simulations to test both the stated assumptions and incorporated several changes to the manuscript to highlight our findings.**

**Firstly, we make a simple modification to remove the globally applied carrying capacity from our model. We then replicate Fig 4c for some representative subsample of the RAG mice (Fig S8B) and observe almost indistinguishable results. We anticipate this outcome, because when sand flies bite, they either regurgitate very few parasites, or far more than is required to infect. Thus, increasing (or in this case removing) the carrying capacity does not change how many bites result in infections, it merely moves previously successful bites even further above the infection threshold. Though we do not consider a reduction to the carrying capacity, our chosen value is informed by actual parasite population sizes reported by Serafim** ***et al.* and carrying capacities that are much smaller than our current value may not properly reflect an *in vivo* system (Serafim *et al.* 2019).**

**Secondly, in order to approximate reduced differentiation efficiency between stages, we modify the model to incorporate a simple, constant population sink at each lifecycle stage. For simplicity, we assume that parasites die (or are otherwise lost) at some constant rate per parasite per day (as detailed in supplementary method S3). We try two different exemplar values, representing a large and a small population sink. Fig S8C and D represent our results. For the smaller population sink, we see almost identical results to those of the original system (Fig. S8A), and although the larger sink gave rise to a quantitative reduction in mean R_0_ we maintain the same qualitative relationship between maximum lifespan and mean R_0_.**

**These results are explained in full at the end of section 1.4 (lines 283-304). Please see also our response to Reviewer 3, Comment 3 for a tangential, but related point.**

2) “Also, I feel uncomfortable with passages such as this one in line 111: “we present expressions bounding implied disease transmission probabilities in a range of hypothetical scenarios”. My distress stems from the fact that the authors do not formulate a true probabilistic model since there is just one source of randomness (N0 in line 117) that propagates deterministically along with the subsequent steps”

**Response: We have made modifications to the introduction to section 1.3 (lines 129-135) to better explain our approach in this section. As the reviewer correctly notes, the population dynamics themselves are deterministic. However, there are two sources of random variation: the initial N_0_ and the chance of a subsequent bite occurring at day 6. We now provide more details regarding this: the model used in section 1.3 is deliberately simplified from that of the full system to make it tractable, such that we may approach it analytically. The full system features additional sources of random variation, namely the use of an exponential random variable to dictate lifespan, inter-bite times are drawn from a gamma distribution, and the probability of biting an infected host at any given bite (which varies between scenarios). We additionally clarify the other sources of randomness at the start of section 1.4 (lines 194 and 196).**

Reviewer 3

1) Clarity is needed on how you derived the retroleptomonad differentiation and replication rates and metacyclic differentiation rates? You quote personal communication with a co-author but this is needs to be explained in greater detail. It is also a discussion point as it raises gaps in our current knowledge.

**Response: We thank the reviewer for identifying this lapse, and sincerely apologise for missing this during our proofreading. We have corrected Table 1 with the actual references to Serafim *et al.* and added an additional statement to our discussion (line 406) to reflect this. Our chosen retroleptomonad replication rate is informed by the population graphs produced by Serafim** ***et al.* and our differentiation rates (from metacyclic to retroleptomonad and vice versa) are chosen to ensure both that the differentiation is completed within a day in the majority of cases, and that the retroleptomonads are eliminated from the population once the ‘dedifferentiation phase’ is complete. However, it is important to note that these are rough estimates – as the reviewer correctly highlights, this represents a gap in our knowledge.**

2) The authors should consider incorporating the findings of Giruad et al. 2019, Communications Biology, into their discussion. They found that heterogeneity in the quality of the transmission (% metacyclics and non-metacyclics deposited) impacts on the course of the infection and transmission back to sand flies. This introduces the possibility of some sand flies acting as super-spreaders. Interestingly, the quality of the transmitted dose can also vary as the (primary) infections mature in the sand fly. This study did not look at the transmission dynamics from second bloodmeal fed flies.

**Response: We thank the reviewer for suggesting the work of Giraud *et al.* for our discussion. We have rewritten and extended a section of our discussion (lines 384-401) which is now devoted to discussing the findings of Giraud *et al.* and their implications for future work on transmission.**

3) Finally, as the interpretation of the author’s model has a lot to do with the lifespan of the infected vector, the authors should incorporate the role of parasite-induced mortality into it. In the discussion, the authors highlight that there is uncertainty in this area because there is a lack of evidence from wild populations. By extension, this argument could apply to all the data within this model. Surely, if sand fly longevity is an important to understand the implications of this model then it should form part of it, particularly, as there is sufficient data in the literature to do this. If this is not possible, the author’s should speculate on how this will influence their model in the discussion

**Response: We feel that this requires careful discussion, and our approach is bipartite.**

1) **We extend our model to incorporate a reduction in lifespan once a sand fly becomes infected. For demonstrative purposes, and inspired by the work of Rogers *et al.* 2007, we chose an exemplar reduction of 20%. To be specific, once a sand fly becomes infected we reduce its remaining lifespan by 20%. We add an additional figure (Fig. S7) to our supplementary figures to represent our results. We observe a reduction in mean R_0_ value, as anticipated. However, we observe no qualitative changes to the behaviour of our model from those represented in Fig. 3.**
2) **We have extended a section to our discussion (lines 338-356) to provide additional details of some of the technical issues faced by lab culturing of sand flies, citing the works of Volf *et al.* (2002) and Volf and Volfova (2011), and also to incorporate our findings regarding parasite induced mortality. Although we can begin to assess the impact of parasite-induced mortality, factors such as bacterial infections arising from lab food and the considerable mortality related to oviposition are unlikely to apply to *in vivo* situations (Killick-Kendrick and Rioux, 2002), and present considerable issues for modelling.**
